# Severe irritability in a critically ill preterm infant: a case of delirium at the neonatal intensive care unit

**DOI:** 10.1590/1980-5764-DN-2022-0046

**Published:** 2023-04-14

**Authors:** Lucas Hirano Arruda Moraes, Vanessa Lisbethe Bezerra Maropo, Ivete Zoboli, Mário Cícero Falcão, Werther Brunow de Carvalho

**Affiliations:** 1 Universidade de São Paulo, Faculdade de Medicina, Departamento de Pediatria, Pós-Graduação em Ciências da Saúde, São Paulo SP, Brazil.; 2 Universidade de São Paulo, Faculdade de Medicina, Departamento de Pediatria, Residente de Neonatologia, São Paulo SP, Brazil.; 3 Universidade de São Paulo, Faculdade de Medicina, Hospital das Clínicas, Instituto da Criança e do Adolescente, Grupo de Dor e Cuidados Paliativos, São Paulo SP, Brazil.; 4 Universidade de São Paulo, Faculdade de Medicina, Departamento de Pediatria, São Paulo SP, Brazil.; 5 Universidade de São Paulo, Faculdade de Medicina, Hospital das Clínicas, Instituto da Criança e do Adolescente, Centro de Terapia Intensiva Neonatal 2, São Paulo SP, Brazil.

**Keywords:** Delirium, Analgesia, Quetiapine Fumarate, Infant, Newborn, Neonatal Abstinence Syndrome, Delirium, Analgesia, Fumarato de Quetiapina, Recém-Nascido, Síndrome de Abstinência Neonatal

## Abstract

Delirium is a common disorder in intensive care units, being associated with greater morbidity and mortality. However, in neonatal intensive care units, delirium is rarely diagnosed, due to the low familiarity of the neonatologist with the subject and the difficulties in the applicability of diagnostic questionnaires. This case report aimed to assess the presence of this disorder in this group of patients and identify the difficulties encountered in the diagnosis and treatment. We report the case of a premature newborn with necrotizing enterocolitis during hospitalization and underwent three surgical approaches. The newborn exhibited intense irritability, having received high doses of fentanyl, dexmedetomidine, clonidine, ketamine, phenytoin, and methadone, without the control of the symptoms. A diagnosis of delirium was then made and treatment with quetiapine was started, with a complete reversal of the symptoms. This is the first case reported in Brazil and the first describing the withdrawal of the quetiapine.

## RESUMO

Delirium é uma síndrome comum em unidades de terapia intensiva, associando-se a maiores morbidade e mortalidade. No entanto, nas unidades de terapia intensiva neonatal, ele raramente é diagnosticado em razão da baixa familiaridade do neonatologista com a suspeita diagnóstica e das dificuldades na aplicabilidade dos questionários diagnósticos. Este relato de caso tem como objetivos mostrar que delirium está presente nesse grupo de pacientes e apontar as dificuldades encontradas no seu diagnóstico e tratamento. Relatamos o caso de um recém-nascido prematuro com enterocolite necrosante, submetido a três abordagens cirúrgicas. O recém-nascido apresentou intensa irritabilidade, tendo recebido altas doses de fentanil, dexmedetomidina, clonidina, cetamina, fenitoína e metadona, sem controle dos sintomas. Em seguida, foi feita a hipótese diagnóstica de delirium e iniciado tratamento com quetiapina, com reversão completa dos sintomas. Este é o primeiro caso notificado no Brasil e o primeiro que descreve a suspensão da quetiapina.

## INTRODUCTION

Delirium is a serious disorder without a fully known pathophysiological mechanism and associated with longer hospital stay, higher morbidity, and mortality in adult and pediatric patients^1,2,3,4,5^. The *Diagnostic and Statistical Manual of Mental Disorders, Fifth Edition* (*DSM-V*) defines delirium as a neurocognitive disorder due to a health condition or its treatment. The symptoms and diagnostic criteria for delirium are:

- Attention disorder and alertness (acute disturbance of consciousness),
- Short time course,
- Fluctuating symptoms, and
- The absence of any other clinical condition that could explain the symptoms^3–6^.

This condition, despite being underdiagnosed, is frequent in critically ill pediatric patients and may be associated with an increase in the length of hospital stay, an increase in the number of days of mechanical ventilation, and an increase in death fourfold^5^.

At neonatal intensive care units (NICUs), delirium is rarely diagnosed. A series of three cases published in 2016 showed significant improvement in newborns who were difficult to sedate and with irritability after using quetiapine for treating delirium, showing that neonatal patients are also affected by this pathology and that the diagnosis and early treatment can improve clinical outcomes^6^. Hereafter, we describe the clinical case of a patient born prematurely who presented with delirium during her hospitalization in the NICU. This case report received the approval of the local ethics committee, and a written consent was given by the parents.

## CASE REPORT

A preterm baby was born by C-section within 31 weeks and 5 days of postmenstrual age, weighing 1,080 g. She had a prenatal diagnosis of Turner’s syndrome and Dandy-Walker’s syndrome and had to be intubated for stabilization, being promptly extubated on the same day at the NICU.

She was stable until 23 days of life (DOL) when she was diagnosed with necrotizing enterocolitis with pneumoperitoneum. An ileal resection with ileostomy was performed on this occasion. She further had two more intestinal perforations, with two other surgical approaches at 51 and 55 DOL. Due to hemodynamic instability and these other complications, she received invasive mechanical ventilation for a total of 33 days and vasoactive drugs for a prolonged period, being kept without enteral feeding during this time.

**Figure 1.**
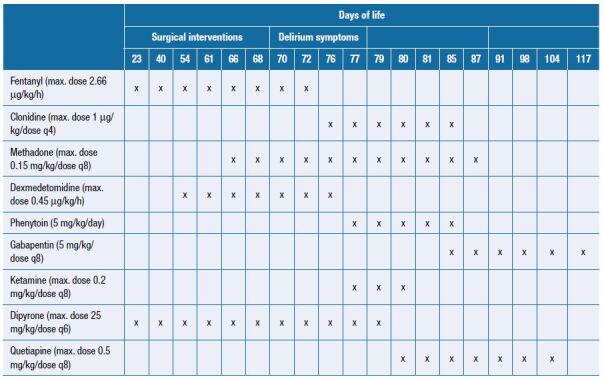
Doses of sedative and analgesic drugs received during the hospitalization.

During hospitalization, aiming a better pain control, she received high doses of sedative and analgesic (Figure 1) medications as continuous intravenous fentanyl (maximum dose – 2.6 μg/kg/h) for a total of 50 days, and continuous intravenous dexmedetomidine (maximum dose – 0.45 μg/kg/h) for a total of 23 days. We further switched fentanyl to intravenous methadone 0.15 mg/kg/dose every 8 h and dexmedetomidine to intravenous clonidine 1 μg/kg/dose every 8 h. After that, she started with periods of agitation, crying, sweating, tachycardia, and intense irritability, which remained unchanged despite non-pharmacological measures. Neonatal sepsis and neonatal opioid-withdrawal syndrome were ruled out (negative cultures and Finnegan score with a maximum score of 6) and then a hypothesis of visceral/neurological pain (maximum Neonatal Infant Pain Scale – NIPS of 4) was made, being initiated intravenous phenytoin 5 mg/kg/day every 12 h and intravenous ketamine 0.2 mg/kg/dose every 8 h. After 3 days, the newborn maintained her clinical condition, with no changes in the Finnegan score and NIPS. After discussing the case with the *Pain and Palliative Care Team,* we performed the Cornell Assessment of Pediatric Delirium (CAPD) with a maximum score of 14 and made the hypothesis of delirium. The assessment was repeated every 3 days according to the international guidelines. We consulted the patient’s parents and, after their consent and a normal record of the QTc interval, we started quetiapine 0.5 mg/kg/dose every 8 h with significant improvement of the clinical status, allowing the return of enteral nutrition after 2 days. After 4 days, we changed phenytoin to enteral gabapentin. Clonidine was discontinued after 7 days and methadone after 10 days.

Fourteen days after starting quetiapine, we began its withdrawal by reducing 0.1 mg/kg/dose every 48–72 h until its total discontinuation. The patient is currently using enteral gabapentin 5 mg/kg/dose every 8 h and is still hospitalized due to postoperative reconstruction of the intestinal tract and probable short bowel syndrome associated with surgical complications.

The infant remains in our service, in intestinal rehabilitation including total parenteral nutrition, waiting for intestinal reconstruction.

## DISCUSSION

Delirium in the neonatal age group is rarely diagnosed, both because of the lack of knowledge by neonatologists and because its symptoms are very similar to opioid-withdrawal syndrome or even acute or chronic pain. The differential diagnosis of delirium in the neonatal period, in addition to the withdrawal syndrome, also includes infections of the central nervous system, exogenous intoxications, and convulsive syndromes. All these clinical conditions were ruled out in the present case.

In the critically ill pediatric population admitted in intensive care units, the incidence of delirium can reach up to 50%, being common in patients using high doses of sedatives such as benzodiazepines and in those children with delayed neurological development^5,7^.

The diagnosis of delirium can be a challenge in pediatrics, especially in the neonatal age group, with diagnostic criteria that overlap between delirium and opioid-withdrawal syndrome^4^. The *European Society of Pediatric and Neonatal Intensive Care* (ESPNIC) recommends that these patients should be evaluated for delirium every 8–12 h (Degree of recommendation: D) through the CAPD (Degree of recommendation: A)^3^. The CAPD is a questionnaire that was created and validated for the pediatric population in 2014, with a sensitivity of almost 95% and a specificity of nearly 80%^8^. It was further translated and validated to Portuguese in 2018^9^.

Despite being the main tool for assessing pediatric delirium and despite having been validated for Portuguese, the CAPD presents some applicability difficulties in newborns, since the first four questions are related to unspecified behavior that can or cannot be present in these populations (Table 1). To solve this problem, it is suggested to use the so-called Anchor-Points to evaluate newborns as young as 4 weeks old^8^. However, there is no validated translation of them in the Portuguese-version of the CAPD yet^9^. In addition, there are still no robust studies in this population to validate this tool^6,10^, especially in those born prematurely.

**Table 1.** Cornell Assessment of Pediatric Delirium tool (only the first four questions)^8^.

Cornell Assessment of Pediatric Delirium – First four questions
1. Does the child make eye contact with the caregiver?
2. Are the child’s actions purposeful?
3. Is the child aware of his/her surroundings?
4. Does the child communicate needs and wants?

Another assessment method for delirium is the Preschool Confusion Assessment for the ICU (PsCAM-ICU). In a recent study published in 2021, Canter et al.^1^ showed a high sensitivity in the use of the method. Their study had the advantage of differentiating newborns from older children. The PsCAM-ICU showed a high negative predictive value and might be useful in excluding this diagnosis in the newborns admitted to NICUs^1^. However, this questionnaire has no translation into Portuguese and further prospective studies using this diagnostic method are still needed.

Our patient had tachycardia, psychomotor agitation, tremors, excessive and inconsolable crying, and food intolerance. In our NICU, the Neonatal Infant Pain Scale is routinely performed in all patients albeit its limitations and confounding factors, with further medical evaluation and analgesic medication titration. When the Finnegan score was applied, the patient did not present a compatible picture of severe opioid-withdrawal syndrome, even so, for many days the condition was managed as such, with a transition from fentanyl to methadone, requiring frequent rescues with morphine and increment of the methadone dose. Furthermore, clonidine was associated to help with the withdrawal of opioid medications. After a differential diagnosis of neuropathic pain, secondary to multiple surgical interventions was made, with intravenous phenytoin being started, also without any improvement in the condition.

Clonidine is an alpha-2 adrenergic agonist that is effective in the treatment of neonatal abstinence syndrome either alone or when associated with other medications^11,12^. It is commonly used in our NICU to aid weaning from opioids in critically ill newborns, particularly those who undergo surgical interventions.

Moreover, phenytoin is an anticonvulsant that is widely used in NICUs for treating neonatal seizures. It can also be used in the treatment of neuropathic pain, being effective in patients with multiple surgical approaches^13^.

As there was no improvement in the clinical condition, we discussed the case with the pain and palliative care team, and the hypothesis of neonatal delirium was made. After searching the literature, we found the case reports by Groves et al.^6^, who successfully used quetiapine in three patients at their NICU, reaching total control of the symptoms. We then initiated intravenous quetiapine for the patient.

Quetiapine is a second-generation atypical antipsychotic medication and has been previously used in pediatric and neonatal populations^14^. Quetiapine is a non-selective antagonist of both dopamine and serotonin receptors and has the Food and Drug Administration (FDA) approval for treating pediatric bipolar disorder. Common adverse reactions are anticholinergic effects, orthostatic hypotension, and torpor. Hopefully, the worst reactions are very rare and include the neuroleptic malignant syndrome, extrapyramidal symptoms, and prolonged QTc. Despite few pharmacodynamic studies in these age groups, its use appears safe and effective in improving and reversing delirium symptoms^14^.

In our case, shortly after starting this medication, it was possible to gradually withdraw the other sedative-analgesic medications and restart the enteral nutrition for the patient.

One limitation is that, although our patient was clearly agitated, we did not perform the *Richmond Agitation-Sedation Scale* as it was only recently cross-cultural validated to Portuguese and still need more studies focusing on the newborn population^15^. Furthermore, we did not evaluate the newborn for hypoactive delirium, as it is of difficult assessment in neonates.

Recent studies and case reports have shown a reduction in the use of sedative-analgesic medications, better control of irritability, reduction in mechanical ventilation days, and reduction in hospital stay after initiating treatment for pediatric or neonatal delirium^1,5–7,10,14,16^. However, we did not find in the current literature what would be the best antipsychotic medication to be used in the neonatal period, due to the lack of prospective studies in this population.

In addition, we did not find the best way to safely withdraw quetiapine. We then performed a gradual withdrawal of 20% of the initial dose every 3 days, until its suspension. In the case presented above, this form of withdrawal does not seem to have been associated with unfavorable events nor with the return of the symptoms.

## CONCLUSIONS

Delirium is associated with an increase in morbidity and mortality in critically ill patients in all age groups. This diagnosis, however, is uncommon in the NICU, with only a few cases reports on this subject in the medical literature. Despite being a frequent pathology, diagnosis is a challenge for neonatologists due to several factors, including the lack of a specific tool for this population, especially for preterm infants. In addition, there is no consensus on how to treat or how to withdraw these antipsychotic agents. More prospective studies to validate diagnostic questionnaires and to assess the best form of treatment are urgently needed to improve the care of these critically ill newborns who are susceptible to developing delirium during the hospital stay.
